# Impact of atropine on changes in choroidal thickness in children with myopia: a meta-analysis of randomized controlled trials

**DOI:** 10.3389/fmed.2025.1678698

**Published:** 2025-10-07

**Authors:** Li Ping Liu, Yun Tang, Jun Na Zhang, Chi Xin Du

**Affiliations:** ^1^Department of Ophthalmology, The Fourth Affiliated Hospital of School of Medicine, and International School of Medicine, International Institutes of Medicine, Zhejiang University, Yiwu, Zhejiang, China; ^2^Department of Ophthalmology, The First Affiliated Hospital, Zhejiang University School of Medicine, Hangzhou, Zhejiang, China

**Keywords:** children with myopia, atropine, choroidal thickness, meta-analysis, myopia progression

## Abstract

**Introduction:**

Atropine is used to treat myopia, and choroidal thickness (ChT) has been suggested as a biomarker for treatment response. However, randomized controlled trials (RCTs) have reported inconsistent results regarding their efficacy. This study aimed to assess the effect of atropine on ChT in children with myopia.

**Methods:**

A systematic review and meta-analysis of RCTs was conducted using PubMed, Cochrane, Embase, and Web of Science databases, including trials registered online, from inception to March 2025. Eligible studies were those that involved patients aged <18 years with myopia treated using atropine sulfate eye drops and reported ChT outcomes. Meta-regression and the Cochrane *I*^2^ test were used to assess heterogeneity, respectively. Publication bias was evaluated using Funnel plots and Egger’s and Begg’s tests. Sensitivity analysis was used to examine the impact of individual studies.

**Results:**

Overall, 11 RCTs involving 1,784 eyes of children with myopia were included. Four doses of atropine (0.01, 0.025, 0.05, and 0.1%) were administered. Subfoveal ChT (SFChT) significantly thickened in the atropine group compared with the control group (placebo or spectacles) during the trial periods [weighted mean difference (WMD): 11.83 μm, 95% confidence interval (CI): 0.88–22.79 μm, *I*^2^ = 98.8%, *p* = 0.000]. Additionally, 0.01% atropine showed the best effect in ChT changes at superior 1 and inferior 1 compared with control. Notably, 0.05% atropine demonstrated the most significantly thickened SFChT (WMD: 25.70 μm, 95% CI: 17.46–33.94 μm), had the best spherical equivalent control (WMD: 0.54 D, 95% CI: 0.38–0.70 D), and had the least axial length elongation (WMD: −0.21 mm, 95% CI: −0.28 to 0.14 mm).

**Conclusion:**

The results showed that atropine may increase ChT than control. Notably, 0.05% atropine may demonstrate the most favorable outcomes for ChT, spherical equivalent, and axial length.

**Systematic Review Registration:**

https://inplasy.com/?s=INPLASY202320027.

## 1 Introduction

Myopia has emerged as a critical public health concern, exhibiting a swift rise in prevalence worldwide. Current projections indicate that approximately 50% of the global population will be impacted by myopia by the year 2050, with 10% facing the potential progression to high myopia (≤−6.00 D). This condition is linked to severe ocular complications that pose a threat to vision, including retinal detachment, glaucoma, and myopic macular degeneration (1, 2). In East Asia, approximately 80–90% of young adults are affected by myopia, highlighting the urgent need for effective interventions (3). The socioeconomic burden of myopia is substantial, encompassing direct healthcare costs, vision rehabilitation expenses, and productivity losses, particularly in pediatric populations where early-onset myopia usually progresses rapidly (4).

Currently recognized approaches to control myopia include optical interventions (orthokeratology, multifocal contact lenses, and defocus-incorporated multiple-segment spectacle lenses) and behavioral modifications (increased outdoor time), which demonstrate 30–60% efficacy in slowing axial elongation (5–7). Many clinicians have recently acknowledged atropine as a safe and efficacious agent for the prevention and management of myopia. However, atropine is believed to exert dose-dependent effects. Although high-dose atropine (1%) has stronger effects in controlling myopia progression than low-dose atropine (0.01%), it is also associated with more side effects and potential risks (photophobia and blurred vision) as well as a more obvious rebound phenomenon after the treatment discontinuation (8). At present, low-concentration atropine has gained prominence as a pharmacological intervention and is most widely used in Asia for children and adolescents with myopia (9–11), showing a 50–60% decrease in its progression while exhibiting minimal adverse effects in multicenter randomized trials (10, 12).

Recent animal and human studies suggest that the choroid plays a major role in slowing myopia progression (13–15). Despite its clinical adoption, the precise mechanism by which atropine exerts its effects remains debated, with emerging evidence suggesting that choroidal thickening is a potential biomarker of treatment response (16). Animal studies have demonstrated that atropine induces choroidal thickening, improves choroidal microcirculation, and reduces scleral hypoxia in myopia management (17, 18). However, data from human randomized controlled trials (RCTs) remain inconsistent, potentially due to variations in treatment duration, dosage, or measurement protocols. Ye et al. (14) reported that 1 and 0.01% atropine concentrations resulted in an increase in choroidal thickness (ChT) among children with myopia, indicating that the choroid is likely an important site for the action of atropine. Yam et al. (19) proposed that the influence of atropine at low concentrations on ChT may exhibit a dose-dependent response during the treatment period. Contrastingly, Kong et al. (20) found no significant effect of 0.01% atropine monotherapy on ChT during a 6-month observation period in school-aged children. Furthermore, only a few meta-analyses have been conducted on atropine and ChT. In a recent meta-analysis, Yang et al. (21) found that atropine significantly increased subfoveal ChT (SFChT) at 6 months. However, their analysis included only four studies. In three of these investigations, the atropine group was administered atropine and orthokeratology treatment, whereas the control group was subjected to orthokeratology treatment alone. Another meta-analysis by Meng et al. (22) demonstrated that the use of 0.01% atropine in children with myopia did not result in a statistically significant difference in SFChT. In their meta-analysis, the control group underwent other treatments, such as orthokeratology lenses, ear acupoint stimulation, and a combination of orthokeratology lenses and atropine. Moreover, RCTs with large sample sizes and extended follow-up periods, such as the myopia outcome study of atropine in children and APP studies, were not included. Therefore, we aimed to perform a meta-analysis, including only RCTs to accurately determine the association between atropine and ChT.

## 2 Materials and methods

### 2.1 Study design

This meta-analysis was performed in accordance with the Preferred Reporting Items for Systematic Reviews and Meta-Analyses guidelines. All research complied with the Declaration of Helsinki, and neither individual patient consent nor ethical reviews were necessary. The methodology for this systematic review was pre-registered on the International Platform of Registered Systematic Review and Meta-analysis Protocols platform (registration number: INPLASY202320027).

### 2.2 Literature search

We conducted a systematic search of several databases, including PubMed, Cochrane, Embase, and Web of Science, covering the period from their inception to March 2025. Medical Subject Headings combined with the free words “myopia,” “atropine,” and “choroidal thickness” were used for the search. We also conducted a supplementary search for relevant studies using ClinicalTrials.gov and Google. Furthermore, we flipped the reference lists of the reported studies to avoid omitting relevant papers.

### 2.3 Inclusion and exclusion criteria

We incorporated all pertinent RCTs that investigated the impact of atropine on ChT in pediatric patients with myopia. The inclusion criteria were as follows: (1) RCTs involving patients aged <18 years; (2) studies in which myopia was diagnosed according to current consensus, specifically defined as a spherical equivalent refraction of ≤0.50 D after cycloplegic autorefraction at baseline (23); (3) studies that reported ChT outcomes following treatment with atropine sulfate eye drops; and (4) if the same research team published multiple studies based on the same research population, the latest and most comprehensive research was selected. Moreover, the exclusion criteria included the following: (1) studies in which valid data could not be obtained; (2) review, meta-analysis, animal studies, protocols, and repeated publications; and (3) studies that evaluated atropine used in conjunction with additional treatment modalities, such as orthokeratology lenses, multifocal soft lenses, and auricular acupoint stimulation.

### 2.4 Data extraction and quality assessment

We used EndNote version X9 (Thomson Reuters) to remove duplicate sections from the articles retrieved earlier. Two independent reviewers examined the titles, abstracts, and full text of the remaining articles according to the predefined inclusion and exclusion criteria. They independently extracted information from the included studies, such as the first author, country, year of publication, study design, sample size, follow-up time, atropine dose, intervention arm, and ChT outcomes. Specifically, ChT was quantified by assessing the distance from Bruch’s membrane to the interface between the choroid and sclera. The Early Treatment Diabetic Retinopathy Study grid was employed to evaluate each scan, dividing the macula into three separate regions. These zones are characterized by circular diameters of 1, 3, and 6 mm, corresponding to the central fovea (subfoveal region), parafoveal areas [superior 1 (S1), inferior 1 (I1), nasal 1 (N1), and temporal 1 (T1)], and perifoveal regions [superior 2 (S2), inferior 2 (S2), nasal 2 (N2), and temporal 2 (T2)], respectively. ChT includes the SFChT, average ChT (AChT), and S1, I1, N1, T1, S2, I2, N2, and T2 ChT. The AChT measured an average ChT of 6 × 6 mm^2^. In case of discrepancy, a third reviewer made the final decision. For studies with multiple intervention arms, we extracted data solely for atropine monotherapy from the intervention group and those for placebo or single-vision glasses from the control group. If a study involved different refractive states, we only extracted data from the myopia group. All data were collected during the atropine treatment period. Changes in ChT were calculated using the following formulas: mean difference (MD) = MD final − MD baseline; Standard deviation^2^ (SD^2^) = SD final^2^ + SD baseline^2^ − 2 × correlation coefficient × SD final × SD baseline. If the SD was not directly provided, it was calculated using the online RevMan calculator based on the standard error or 95% confidence interval (CI).

The quality of the included RCTs was assessed using the risk-of-bias tool developed by the Cochrane Collaboration. This tool encompasses the following seven distinct domains: generation of random sequences, concealment of allocation, blinding of both participants and personnel, blinding of outcome evaluation, incomplete data regarding outcomes, selective reporting, and the presence of other biases. Two reviewers evaluated the potential for bias across each domain, classifying it into three categories as follows: “low,” “high,” or “unclear.”

### 2.5 Data synthesis and analysis

The Stata MP 15 software was used for statistical analysis. We examined the impact of atropine on ChT in pediatric patients with myopia by determining the weighted mean difference (WMD) and 95% CI for subsequent analyses. The Cochrane *I*^2^ test was used to assess heterogeneity. An *I*^2^ ≥ 50% indicated significant heterogeneity, prompting the application of a random-effects model for the pooled data. A fixed-effects model was utilized if the *I*^2^ value was <50%. Funnel plots, along with Egger’s and Begg’s tests, were employed to assess the risk of publication bias. Sensitivity analysis was conducted to evaluate the influence of each study on the overall results by excluding the included articles individually. Meta-regression analysis was conducted to identify the origins of heterogeneity. Statistical significance was established at *p* < 0.05.

## 3 Results

### 3.1 Search results

**Figure 1:**
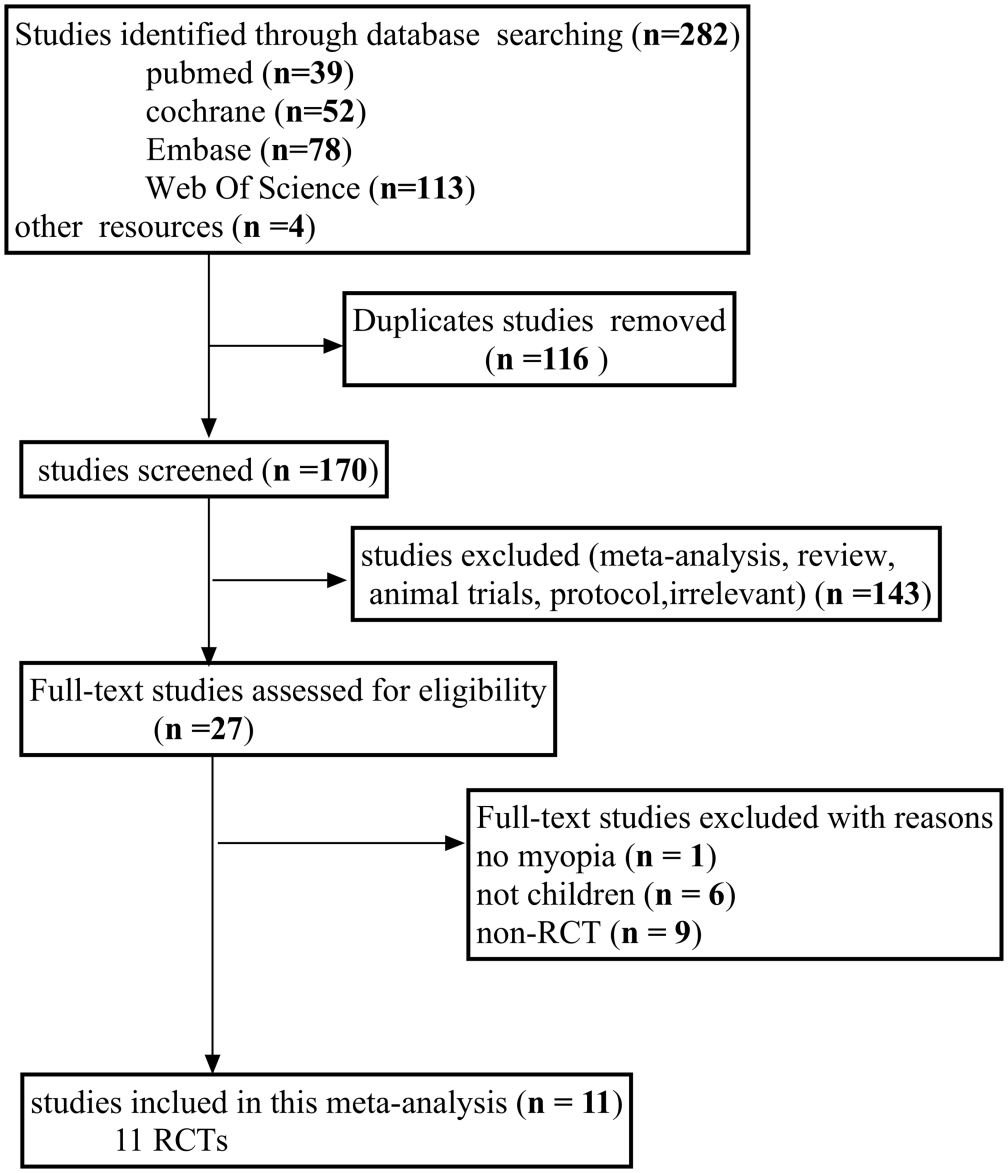
Flow diagram of the literature search process following the PRISMA guidelines. PRISMA, Preferred Reporting Items for Systematic Reviews and Meta-Analyses.

In total, 286 studies were identified through an online search of relevant articles published until March 2025. After screening the titles and abstracts of the remaining studies, 116 duplicate articles were removed, and 143 irrelevant articles were eliminated. Ultimately, 11 RCTs were incorporated into the meta-analysis following a comprehensive evaluation of the full texts (Figure 1).

### 3.2 Study characteristics and quality assessment

**Figure 2:**
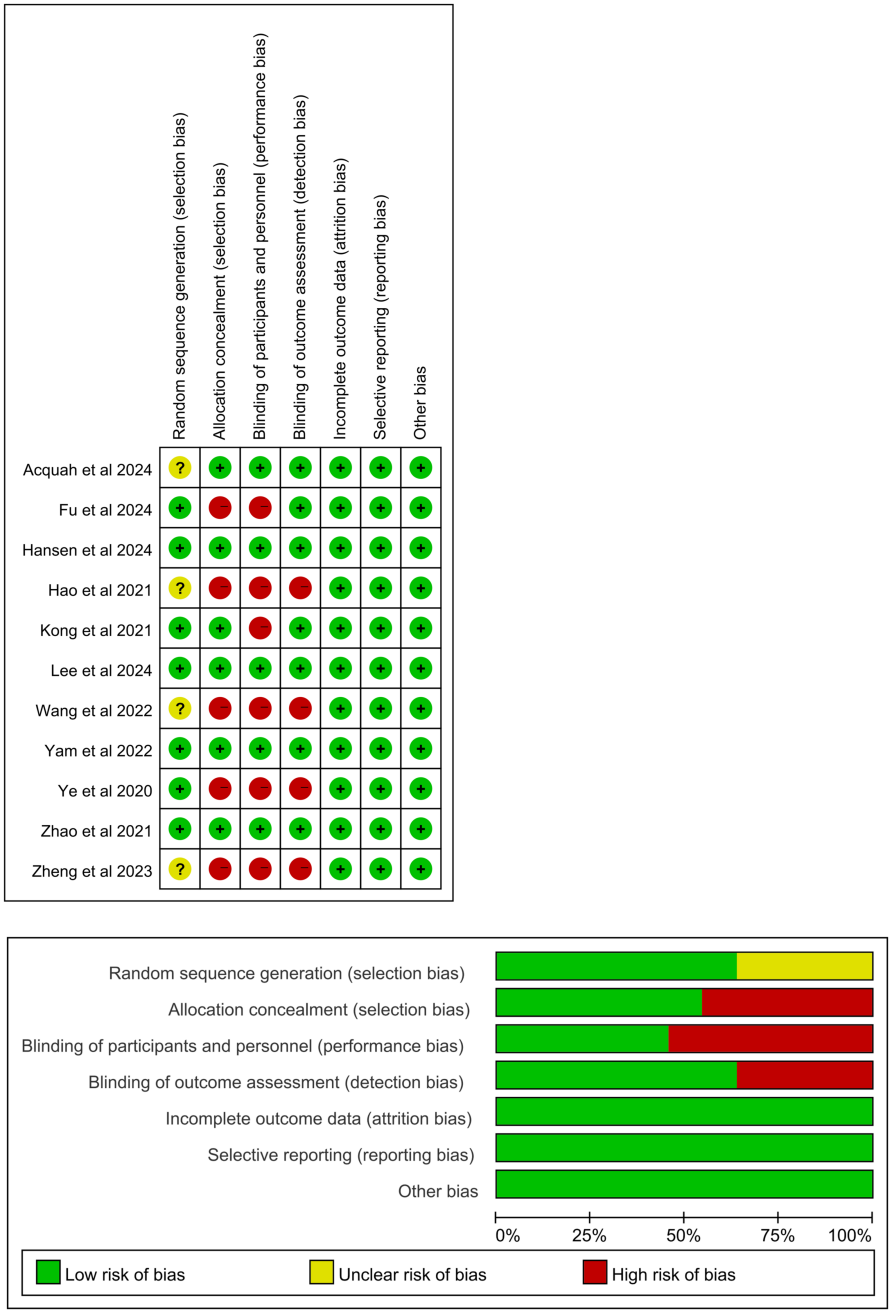
Risk of publication bias assessment of the included studies.

Table 1 summarizes the baseline characteristics of the 11 RCTs involving 1,784 eyes included in this meta-analysis (16, 19, 20, 24–31). All included articles were published between 2020 and 2024. Atropine was administered once every night in all studies (Supplementary Table 1). The participants in the 11 studies were from various countries: Mainland China in seven studies, and Hong Kong, Australia, Denmark and Ireland in one study each. Seven studies described fixed examination times, whereas the other four did not. The duration of atropine treatment varied across the 11 studies. Among them, four lasted 24 months, one lasted 12 months, four lasted 6 months, and two lasted <6 months. However, only seven studies included control groups (placebo or spectacles). Figure 2 illustrates the findings regarding the risk of publication bias in the included RCTs. All 11 studies presented a low-to-moderate risk of publication bias.

**Table 1:** Characteristics of the included studies at baseline in the meta-analysis.

Study (author year)	Study design	Country	Research groups and sample sizes	Follow-up time (month)	Enrolled ages (y)	Measure time	AL (mm) mean (SD)	SE (D) mean (SD)	Choroidal thickness (μm) mean (SD)
Yam et al. 2022 (19)	RCT	Hong Kong	0.05% A (81) 0.025% A (80) 0.01% A (86) Placebo (69)^a^	4, 8, 12, 16, 20, 24	4–12	3 p.m.–5 p.m.	24.85 (0.90) 24.86 (0.95) 24.70 (0.99) 24.82 (0.97)	−3.98 (1.69) −3.71 (1.85) −3.77 (1.85) −3.85 (1.95)	242.37 (51.07) 250.36 (56.97) 244.13 (58.54) 238.35 (57.14)
Kong et al. 2021 (20)	RCT	China	0.01% A (50) 0.01% A + AAS (50)	1, 3, 6	7–12	NG	24.48 (0.76) 24.30 (0.86)	−2.25 (1.14) −2.14 (1.27)	233.45 (22.95) 233.83 (28.68)
Ye et al. 2020 (16)	RCT	China	0.01% A (87) 1% A (98)	0.25, 3, 6	6–12	10 a.m.–3 p.m.	24.27 (0.74) 24.34 (0.82)	−2.16 (1.10) −2.12 (1.09)	218 (39)^c^ 214 (45)^c^
Zhao et al. 2021 (24)	RCT	China	0.01% A + OK (39) 0.01% A (42) Placebo + OK (36) Placebo + spectacles (37)	1	8–12	3 p.m.–6 p.m.	24.78 (0.98) 24.90 (0.78) 24.69 (0.63) 24.86 (0.72)	−3.12 (1.20) −3.01 (1.22) −2.74 (1.06) −3.25 (1.10)	263.17 (46.55) 251.12 (44.76) 266.74 (57.50) 258.05 (52.34)
Hao et al. 2021 (25)	RCT	China	0.01% A (22) OK (24) 0.01% A + OK (21)	1, 6, 12	8–12	NG	24.91 (0.61) 25.17 (0.52) 25.29 (0.56)	−3.62 (0.57) −3.66 (0.60) −4.07 (0.74)	240.64 (19.93) 236.83 (16.78) 235.14 (20.33)
Wang et al. 2022 (26)	RCT	China	0.01% A (21) Spectacles (18)	1, 3	6–14	12 a.m.–3 p.m.	24.45 (1.06) 24.70 (0.93)	−2.38 (1.46) −2.36 (1.87)	249.98 (38.26) 229.78 (46.73)
Lee et al. 2024 (27)	RCT	Australia	0.01% A (89) Placebo (30)	24	6–16	NG	24.6 (24.2–25.2)^d^ 24.8 (24.3–25.4)^d^	−3.13 (−4.13 to −2.38)^d^ −3.50 (−4.50 to −2.63)^d^	252.4 (215.10) 256.1 (125.85)
Fu et al. 2024 (28)	RCT	China	0.01% A (42) LLRL (45)	3, 6	6–12	NG	24.52 (0.82) 24.33 (0.81)	−2.28 (1.04) −2.43 (1.13)	286.62 (38.93) 297.89 (51.04)
Hansen et al. 2024 (29)	RCT	Denmark	0.01% A (32) 0.1% A + 0.01% A (33)^b^ Placebo (32)	3, 6, 24	6–12	9:30 a.m.–1:30 p.m.	24.56 (0.78) 24.48 (0.86) 24.41 (0.90)	−2.97 (1.13) −3.0 (1.59) −3.07 (1.04)	260 (66.70) 240 (67.20) 244 (65.10)
Kobia-Acquah et al. 2024 (30)	RCT	Ireland	0.01% A (252) Placebo (122)	12, 18, 24	6–12	9 a.m.–4 p.m.	24.83 (1.06) 24.82 (1.07)	−3.18 (−4.46 to −2.03)^d^ −3.40 (−4.25 to −1.90)^d^	240.5 (61.5) 229.7 (55.8)
Zheng et al. 2023 (31)	RCT	China	0.01% A (124) Spectacles (122)	3, 6	NG	12:30 a.m.–3:30 p.m.	24.92 (1.46) 24.83 (0.87)	−4.05 (2.93) −4.11 (2.50)	224.50 (50.82) 227.02 (49.43)

SFChT, subfoveal choroidal thickness; OK, orthokeratology; NG, not given; RCT, randomized controlled trial; SD, standard deviation; A, atropine; LLRL, low-level red light; SE, spherical equivalent; AL, axial length; AAS, auricular acupoint stimulation.

a Change to 0.05% atropine treatment after 12 months.

b 0.1% atropine use at first 6 months, then change to 0.01% atropine after 6 months.

c Ye et al. (16): choroidal thickness measured the average choroidal thickness (area 6 × 6 mm^2^). Other studies: choroidal thickness measured subfoveal choroid thickness.

d Mean median [interquartile range (IQR)].

### 3.3 Results of the meta-analysis

#### 3.3.1 Meta-regression analysis

A meta-regression analysis was conducted to identify the source of heterogeneity based on different follow-up periods, types of ChT, and doses of atropine, race, ChT measure time, and the instrument of optical coherence tomography. The doses of atropine (*p* = 0.047) were found to be the source of heterogeneity.

#### 3.3.2 Changes in choroidal thickness among children with myopia in the atropine versus control group

**Figure 3:**
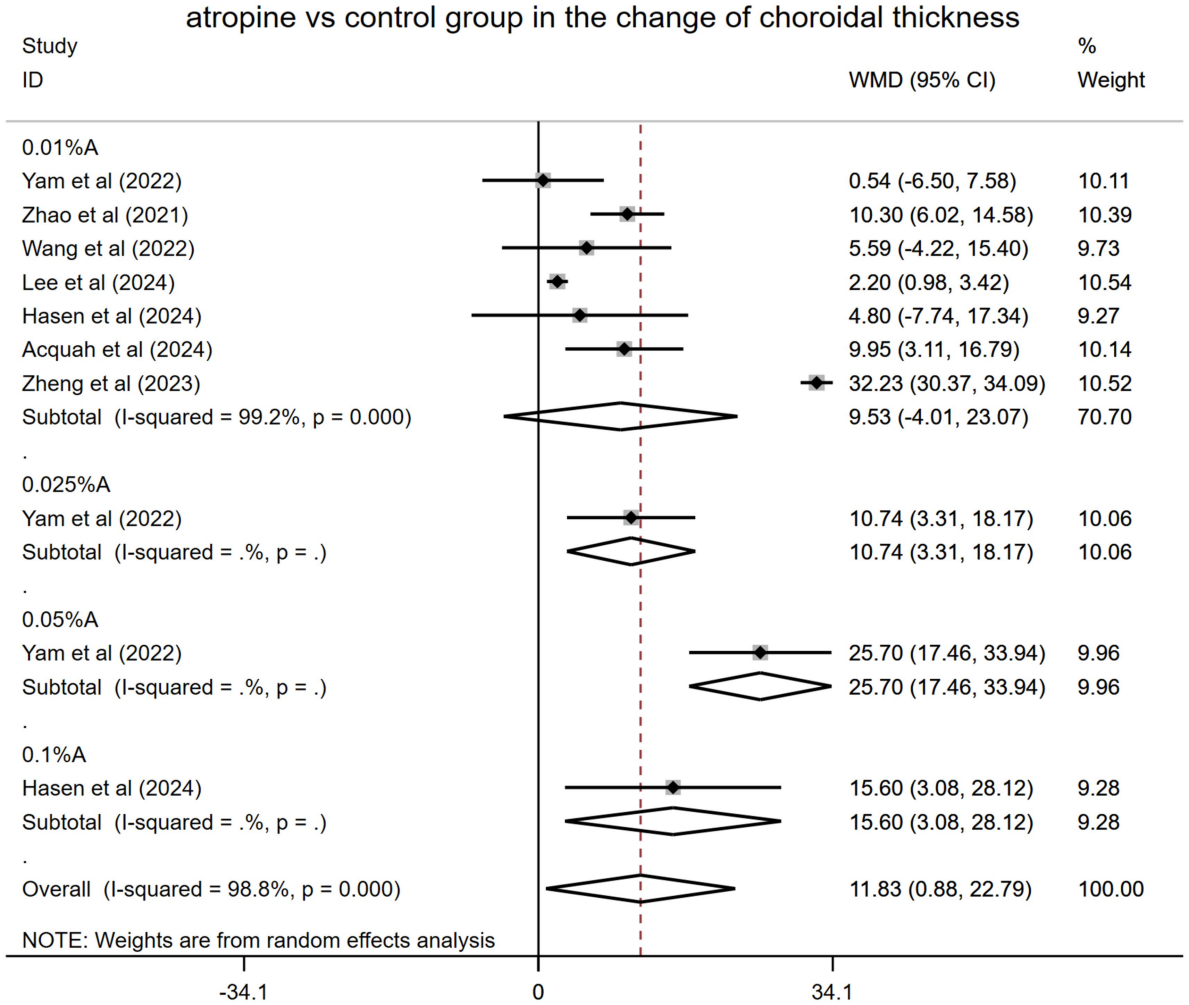
Changes observed in choroidal thickness in the subgroup analysis in the atropine versus control group. A, atropine, WMD, weighted mean difference.

Among the 11 RCTs, seven included a control group (placebo or spectacles), whereas the other four did not. Data from the seven RCTs (originating from four Asian and three non-Asian countries) involving 1,270 eyes were pooled (Figure 3). The final follow-up results for each RCT were used in the analysis. Specifically, the control group was exclusively composed of individuals who received either a placebo or corrective spectacles. Yam et al. (19) had a 1-year follow-up period, after which the control group began receiving 0.05% atropine treatment. SFChT was the ChT type measured in all seven studies. The merged result showed that SFChT was significantly thicker in the atropine group than in the control group during the trial periods (WMD: 11.83 μm, 95% CI: 0.88–22.79 μm, *I*^2^ = 98.8%, *p* = 0.000).

However, in the subgroup analysis, the effects of different atropine doses varied as follows: WMD: 9.53 μm, 95% CI: −4.01 to 23.07 μm, *I*^2^ = 99.2%, *p* = 0.000 for 0.01% atropine; WMD: 10.74 μm, 95% CI: 3.31–18.17 μm for 0.025% atropine; WMD: 25.70 μm, 95% CI: 17.46–33.94 μm for 0.05% atropine; and WMD: 15.60 μm, 95% CI: 3.08–28.12 μm for 0.1% atropine. The impact of 0.01% atropine on ChT alterations was the worst, with no statistical significance. Other doses of atropine (0.025, 0.05, and 0.1%) had better and statistically significant effects. The 0.05% atropine group showed the greatest SFChT change, which was significantly thickened (Figure 3).

Sensitivity analyses performed across the included studies (Supplementary Figure 2) demonstrated consistent stability in the pooled effect estimates.

The methodological evaluation of publication bias incorporated a funnel plot visualization (Supplementary Figure 2), which revealed mild asymmetry potentially attributable to multiple factors, including selective publication, inter-study effect size variability, and random variation. Quantitative assessment using Egger’s (*t* = 0.31, *p* = 0.76) and Begg’s (*z* = 0.36, *p* = 0.72) tests yielded non-significant results, indicating no statistically detectable publication bias within the seven-study cohort.

#### 3.3.3 Effect of atropine on changes in choroidal thickness among children with myopia from baseline to the final treatment period (self-control)

**Figure 4:**
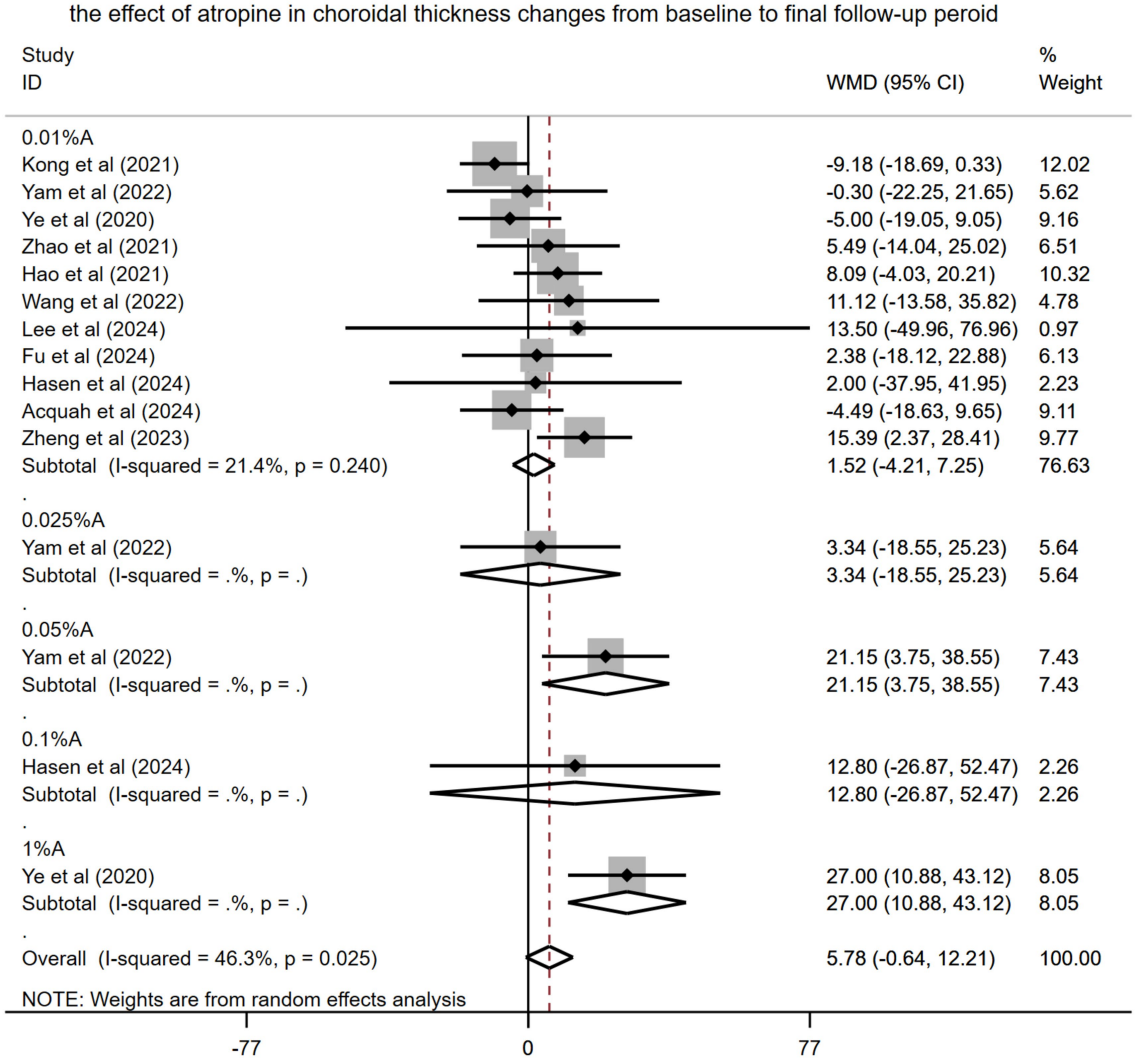
Effects of atropine on changes in choroidal thickness among children with myopia from baseline to the final treatment period (self-control). A, atropine, WMD, weighted mean difference.

Eleven studies involving 1,139 children with myopia were incorporated into this meta-analysis. Ye et al. (14) used the AchT as a measure of ChT, whereas other studies used the SFChT. The pooled result found that the ChT became thickened from baseline to the final follow-up period after using atropine (WMD: 5.78 μm, 95% CI: −0.64 to 12.21 μm, *I*^2^ = 46.3%, *p* = 0.02) (Figure 4). However, the difference did not reach statistical significance.

Subgroup analysis of the pooled results revealed that various concentration of atropine differed in their impact on patient outcomes (WMD: 1.52 μm, 95% CI: −4.21 to 7.25 μm for 0.01% atropine, WMD: 3.34 μm, 95% CI: −18.55 to 25.23 μm for 0.025% atropine, WMD: 21.15 μm, 95% CI 3.75–38.55 μm for 0.05% atropine, WMD: 12.80 μm, 95% CI: −26.87 to 52.47 μm for 0.1% atropine, and WMD: 27.0 μm, 95% CI: 10.88–43.12 μm for 1% atropine). However, only 0.05 and 1% atropine had a significant effect on ChT.

Sensitivity analyses demonstrated robust consistency across methodological evaluations (Supplementary Figure 3). Funnel plot analysis (Supplementary Figure 4) was employed to assess the risk of publication bias within the 11 studies included in the review, with visual interpretation revealing symmetrical distribution patterns. Quantitative assessment using Egger’s (*t* = 1.11, *p* = 0.29) and Begg’s (*z* = 0.30, *p* = 0.77) tests confirmed the lack of statistically meaningful publication bias within the studied cohort.

#### 3.3.4 Effects of 0.01% atropine on changes in choroidal thickness among children with myopia

##### 3.3.4.1 0.01% atropine versus control

The results of seven RCTs that measured SFChT as the ChT were merged. In the subgroup analysis of races, the aggregated results indicated that 0.01% atropine had a more favorable impact on ChT within the Asian population than with the non-Asian population. Nevertheless, the difference did not reach statistical significance (WMD: 12.42 μm, 95% CI: −4.59 to 29.43 μm and WMD: 9.53 μm, 95% CI: −4.01 to 23.07 μm, respectively) (Table 2).

**Table 2:** Subgroup analyses of efficacy outcomes in the changes in choroidal thickness with 0.01% atropine.

The changes of choroidal thickness (um)									
0.01% atropine vs control group					0.01% atropine self-control				
Subgroups	No. of studies	Pooled WMD (95% CI)	*p*-value	*I*^2^ (%)	Subgroups	No. of studies	Pooled WMD (95% CI)	*p*-value	*I*^2^ (%)
Overall	7	9.53 (−4.01 to 23.07)	*p* > 0.05	99.2	Overall	10	2.56 (−0.76 to 5.88)	*p* > 0.05	0
Races					Races				
Asian population Non-Asian population	4 3	12.42 (−4.59 to 29.43) 9.53 (−4.01 to 23.07)	*p* > 0.05 *p* > 0.05	98.1 99.2	Asian population Non-Asian population	7 3	2.23 (−3.32 to 7.78) 4.63 (−8.40 to 17.65)	*p* > 0.05 *p* > 0.05	43.6 0
Treatment period					Treatment period				
1 m 3 m 6 m 12 m 24 m	2 4 3 2 2	6.09 (−2.69 to 14.87) 9.03 (−4.63 to 22.70) 13.27 (−10.54 to 37.09) 3.95 (−2.13 to 10.04) 5.33 (−2.12 to 12.78)	*p* > 0.05 *p* > 0.05 *p* > 0.05 *p* > 0.05 *p* > 0.05	82.0 94.3 97.5 41.9 79.5	1 m 3 m 6 m 12 m 24 m	4 5 5 3 3	2.24 (−4.49 to 8.96) 3.08 (−3.89 to 10.04) 1.55 (−4.56 to 7.66) 3.77 (−4.69 to 12.23) 3.46 (−8.21 to 15.14)	*p* > 0.05 *p* > 0.05 *p* > 0.05 *p* > 0.05 *p* > 0.05	0 11.9 59.7 0 0

WMD, weighted mean difference; CI, confidence interval; m, month.

The aggregated results in the subgroup analysis of the treatment period indicated that the peak effect of 0.01% atropine versus control group in ChT was at 6 months (WMD: 13.27 μm, 95% CI: −10.54 to 37.09 μm); however, it was not statistically significant (Table 2).

##### 3.3.4.2 0.01% atropine self-control

The results of 10 RCTs that measured SFChT as the ChT were merged. Table 2 shows the subgroup analysis of the effect of 0.01% atropine on SFChT changes among children with myopia according to race. The findings did not reach statistical significance.

A subgroup analysis was conducted to evaluate the impact of 0.01% atropine on SFChT changes among children with myopia across different treatment periods. The findings were WMD: 2.24 μm, 95% CI −4.49 to 8.96 μm at 1 month; WMD: 3.08 μm, 95% CI: −3.89 to 10.04 μm at 3 months; WMD: 1.55 μm, 95% CI: −4.56 to7.66 μm at 6 months; WMD: 3.77 μm, 95% CI: −4.69 to 12.23 μm at 12 months, and WMD: 3.46 μm, 95% CI: −8.21 to 15.14 μm at 24 months. However, no statistical significance was observed. Table 2 present the pooled outcomes.

#### 3.3.5 Effects of atropine versus control treatment on changes in choroidal thickness among children with myopia at different positions

**Figure 5:**
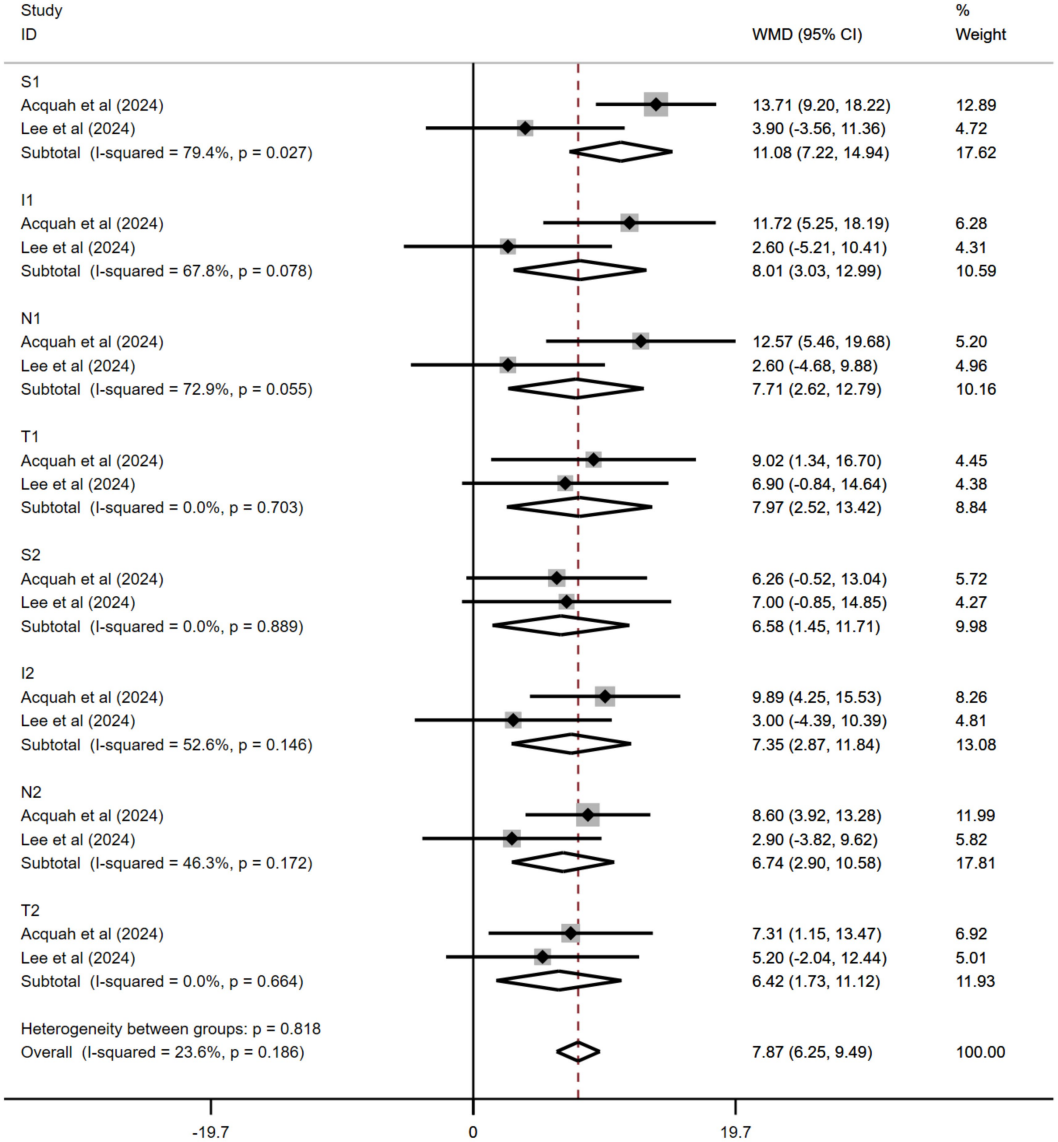
Effects of atropine versus control treatment on changes in choroidal thickness among children with myopia at different positions. S, superior, I, inferior, N, nasal, T, temporal, WMD, weighted mean difference.

Only studies by Kobia-Acquah et al. (30) and Lee et al. (27) were pooled. In both studies, the follow-up period was 24 months, and all patients received 0.01% atropine. The pooled data showed significantly thicker ChT in the 0.01% atropine group than in the control group during the trial periods (WMD: 7.87 μm, 95% CI: 6.25–9.49 μm, *I*^2^ = 23.6%, *p* = 0.186) (Figure 5). These effects varied at different positions. S1 and I1 had the best effect on ChT changes (WMD: 11.08 μm, 95% CI: 7.22–14.94 μm and WMD: 8.01 μm, 95% CI: 3.03–12.99 μm, respectively). The effects of other positions included WMD: 7.71 μm, 95% CI: 2.62–12.79 μm at N1; WMD: 7.97 μm, 95% CI: 2.52–13.42 μm at T1; WMD: 6.58 μm, 95% CI: 1.45–11.71 μm at S2; WMD: 7.3 5 μm, 95% CI: 2.87–11.84 μm at I2; WMD: 6.74 μm, 95% CI: 2.90–10.58 μm at N2; and WMD: 6.42 μm, 95% CI: 1.73–11.12 μm at T2. All results were statistically significant.

#### 3.3.6 Effects of atropine versus control treatment on changes in spherical equivalent among children with myopia

**Figure 6:**
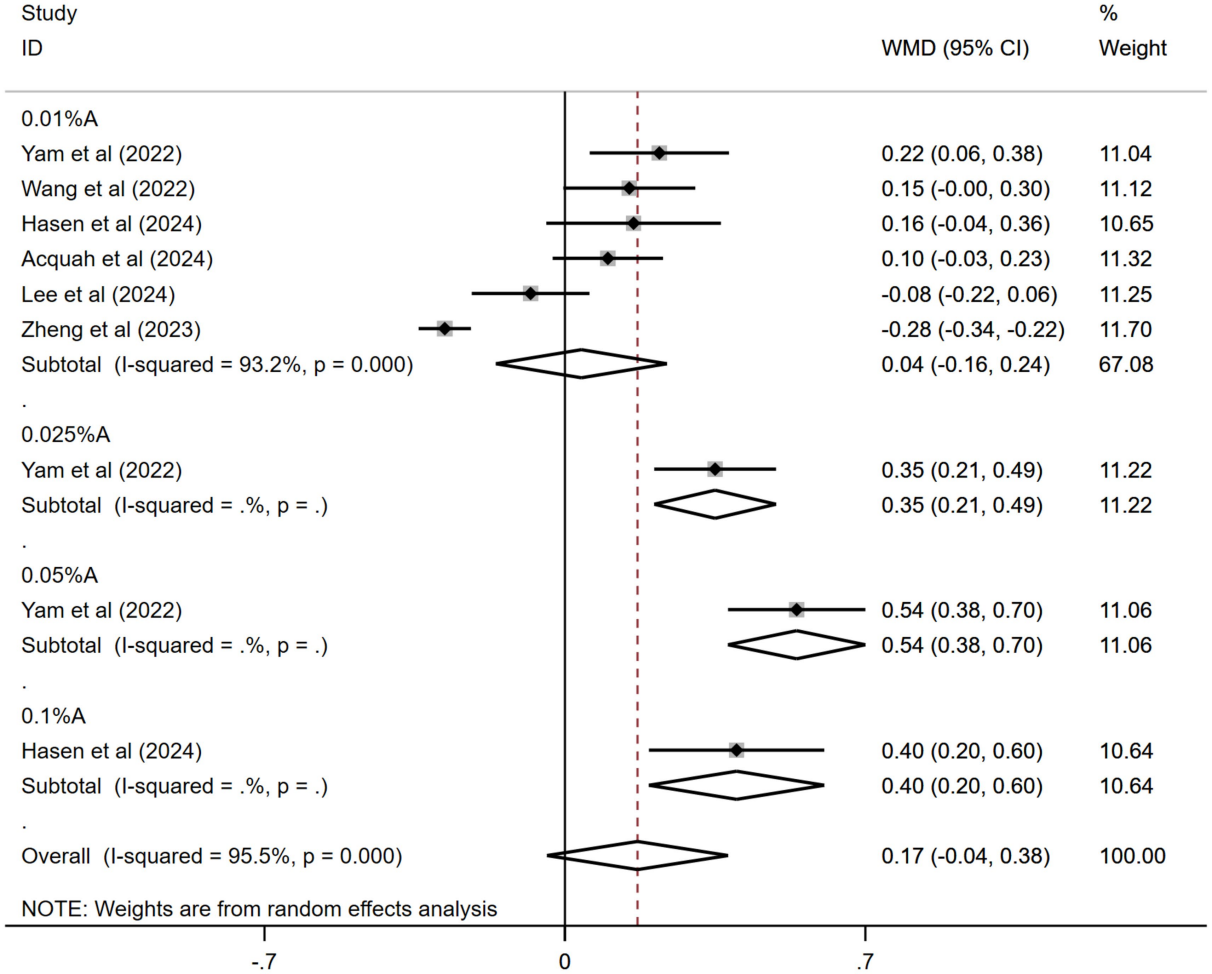
Effects of atropine versus control treatment on changes in spherical equivalent among children with myopia. A, atropine, SE, spherical equivalent, WMD, weighted mean difference.

The results from six of the 11 studies were pooled. Figure 6 shows the combined outcomes. The pooled data showed a greater spherical equivalent change in the atropine group than in the control group during the trial period (WMD: 0.17 D, 95% CI: −0.04 to 0.38 D, *I*^2^ = 95.5%, *p* = 0.000). Nonetheless, the findings did not achieve statistical significance.

In the subgroup analysis, 0.05% atropine had the most spherical equivalent change (WMD: 0.54 D, 95% CI: 0.38–0.70 D). The results for other atropine concentrations were WMD: 0.35 D, 95% CI: 0.21–0.49 D for 0.025% atropine; WMD: 0.40 D, 95% CI: 0.20–0.60 D for 0.1% atropine; and WMD: 0.04 D, 95% CI: −0.16 to 0.24 D for 0.01% atropine. Only the effects of 0.05, 0.025, and 0.1% atropine on spherical equivalent changes were statistically significant.

The sensitivity analyses performed across the included studies (Supplementary Figure 5) demonstrated consistent stability in the pooled effect estimates. Funnel plot analysis (Supplementary Figure 6) was utilized to assess the risk of publication bias within the six studies that were incorporated. The Egger’s test (*t* = 4.98, *p* = 0.002) suggested a publication bias, whereas the Begg’s test (*z* = 1.36, *p* = 0.18) did not.

#### 3.3.7 Effects of atropine versus control treatment on changes in axial length among children with myopia

**Figure 7:**
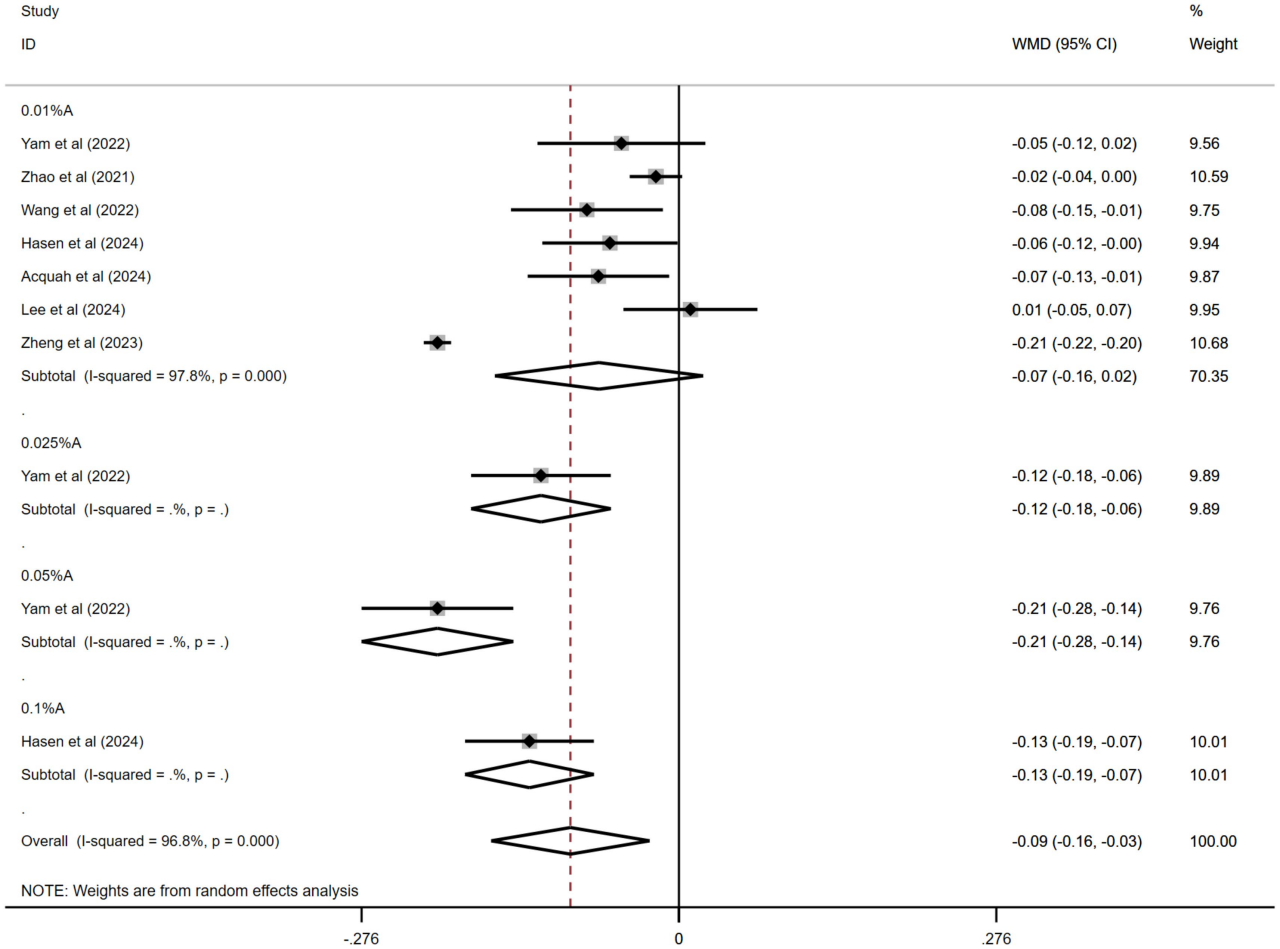
Effects of atropine versus control treatment on changes in axial length among children with myopia. A, atropine, AL, axial length, WMD, weighted mean difference.

Seven of the 11 studies were pooled. Figure 7 shows the combined outcomes. The aggregated results revealed significantly less axial length (AL) elongation in the atropine group than in the control group during the trial periods (WMD: −0.09 mm, 95% CI: −0.16 to −0.03 mm, *I*^2^ = 96.8%, *p* = 0.000).

In the subgroup analysis, 0.05% atropine had the least AL elongation (WMD: −0.21 mm, 95% CI: −0.28 to −0.14 mm). The results for other atropine concentrations were WMD: −0.12 mm, 95% CI: −0.18 to −0.06 mm for 0.025% atropine; WMD: −0.13 mm, 95% CI: −0.19 to −0.07 mm for 0.1% atropine; and WMD: −0.07 mm, 95% CI: −0.16 to −0.02 mm for 0.01% atropine. Only the effects of 0.05, 0.025, and 0.1% atropine on AL changes were statistically significant.

The sensitivity analyses performed across the included studies (Supplementary Figure 7) demonstrated consistent stability in the pooled effect estimates. A comprehensive evaluation of publication bias was conducted using funnel plot visualization (Supplementary Figure 8) across the seven studies included. Quantitative assessment using Egger’s (*t* = 1.9, *p* = 0.09) and Begg’s (*z* = 0.00, *p* = 1.0) tests yielded non-significant results, indicating no statistically detectable publication bias among the seven studies.

## 4 Discussion

Atropine is a muscarinic acetylcholine receptor antagonist commonly considered a safe and efficacious option for controlling myopia progression, particularly at low concentrations (9, 12, 32, 33). Despite this, the specific mechanism by which atropine exerts its effects on myopia remains poorly understood. ChT changes are considered important for controlling myopia using atropine. Nevertheless, results regarding these changes vary significantly among human studies (34–37). While some trials report 10–20 μm choroidal thickening with 0.01% atropine (26), others show no significant changes (20). Meta-analyses evaluating the relationship between atropine and ChT are limited and show inconsistent results. No existing meta-analysis has synthesized RCT evidence on this relationship, creating uncertainty regarding whether ChT modulation contributes to the anti-myopic effects of atropine. Consequently, this systematic review and meta-analysis addressed the following critical gaps by: (1) quantifying the magnitude and consistency of choroidal responses across RCTs (three studies had seven domains with a low risk according to the Cochrane risk assessment), (2) exploring dose–response relationships and the effects of different positions, and (3) evaluating its potential as a predictive biomarker that will optimize patient selection and inform mechanistic research into anti-myopia therapies.

Our meta-analysis included patients treated with 0.01, 0.025, 0.05%, or 0.1% atropine. The outcomes indicated that atropine demonstrated a greater efficacy in increasing the SFChT thickness than the control group, as well as concentration-dependent effects of atropine on SFChT changes. Particularly, 0.05% atropine appeared to exhibited the most pronounced outcomes in increasing SFChT (WMD: 25.70 μm, 95% CI: 17.46–33.94 μm), controlling AL elongation (WMD: −0.21 mm, 95% CI: −0.28 to −0.14 mm), and reducing the spherical equivalent (WMD: 0.54 D, 95% CI: 0.38–0.70 D) than the lower (0.01 and 0.025%) and higher (0.1%) concentrations in the available studies. This result aligned with the outcomes of Ha et al. (9) and Wang et al. (38), who both presented that 0.05% atropine had the best rank probability in terms of preventing the overall progression of myopia. However, only a few studies have been conducted on 0.05, 0.025, and 0.1% atropine; therefore, further research with larger sample sizes and more high-quality RCTs is needed to confirm these preliminary findings.

We found that 0.01% atropine had no statistically significant impact on ChT changes compared with the control or atropine self-control at different treatment nodes during the trial period. The findings indicated that the peak time for the impact of 0.01% atropine on ChT changes was 6 months compared with that in the control group. Although the findings did not achieve statistical significance, they suggest that doctors consider discontinuing the use of 0.01% atropine in children with myopia if the treatment impact remains poor at 6 months. Several factors may have contributed to the reduced effectiveness of atropine in myopia after 6 months. One reason for this is that, as the eye grows naturally, AL elongation limits ChT. Although atropine may increase ChT, children with rapid AL growth also exhibit choroidal thinning. Therefore, eyeball elongation can counteract atropine-induced choroidal thickening. However, its underlying mechanisms remain unknown. One accepted hypothesis is that atropine treatment causes choroidal vasculature expansion and increases blood flow through nitric oxide-mediated changes in the choroid and surrounding smooth muscles (39–41). Therefore, we speculate that the 6-month duration represents the limit to choroidal vessel dilation. Since 0.01% atropine is the most widely used—and is the only commercially available option in some countries, such as China—research on atropine at other concentrations (such as 0.05, 0.025, and 0.1%) is limited to its assessment of myopia, with even fewer studies examining its effects on the choroid. Consequently, further studies focusing on other doses of atropine for myopia control are required.

Our meta-analysis demonstrated superior thickening effects of atropine in the parafoveal and perifoveal regions compared with the fovea (SFChT WMD: 5.33 μm) at 24 months after treatment, with maximal response observed in S1 and I1 sectors. This spatial heterogeneity may stem from regional variations in choroidal vascular density, as the perifoveal area contains higher concentrations of vessels than the relatively avascular foveal center. The enhanced sensitivity of the vertical regions (S1/I1 sectors) may be related to the asymmetric distribution of muscarinic receptors at the choroid-scleral interface. In contrast, the relative resistance of the foveal region can be attributed to its unique structural characteristics. The dense cone photoreceptor population of the fovea and Müller cell cones creates a specialized metabolic microenvironment with tight regulation of blood flow. This intrinsic homeostasis may limit pharmacological responsiveness compared with the peripheral regions, where vascular compliance is greater. Additionally, the role of the foveal choroid in maintaining optical transparency through a precise fluid balance may necessitate stricter regulatory mechanisms against thickness variations. Therefore, peripheral ChT of the macular fovea could act as an innovative biomarker for evaluating therapeutic efficacy in myopia control, as its reactivity could better capture early choroidal changes than traditional foveal measurements.

The overall heterogeneity observed in the study was significantly elevated, and the meta-regression analysis pinpointed atropine dose as the contributing factor to this heterogeneity. Consequently, we conducted a subgroup analysis categorized by atropine dose. Despite this, the subtotal heterogeneity remained notably high. The considerable heterogeneity noted among the different studies may be indicative of discrepancies in the populations examined, protocols employed for interventions, and methods of outcome evaluation. While a subgroup analysis was performed, its validity is limited by the relatively few studies that were incorporated. Therefore, the aggregated effect estimates must be regarded with caution owing to this significant variability.

Nonetheless, this meta-analysis has some limitations. First, the results of our analysis may have been limited by the small sample size. Therefore, increasing the quantity and caliber of RCTs incorporated in this research—currently featuring only 11 studies—is essential. Only seven studies had a qualified control group. While most of the studies used a concentration of 0.01% atropine, only a few used other concentrations. Therefore, additional research on different doses of atropine, particularly on low doses other than 0.01%, is essential. Second, significant variability was observed in the results. Although subgroup analyses were performed based on different concentrations, treatment times, ethnicities, and choroidal locations, significant heterogeneity was observed. Baseline age, myopia, atropine preparations, examination instruments, choroidal measurement methods, and choroidal calculation techniques can also affect heterogeneity. Third, fluctuations in ChT throughout the day may affect the measurements and results (42). The absence of standardized inspection times in the included studies may have introduced errors into the study. Finally, treatment duration in the incorporated studies was short, with seven of the 11 studies having a treatment duration of ≤6 months.

In conclusion, atropine may increase ChT compared with controls (placebo and spectacles). Notably, 0.05% atropine may demonstrated the most favorable outcomes in terms of ChT, spherical equivalent, and AL. However, the current evidence is limited. Therefore, more robust RCTs are needed to assess different doses and improve clinical guidelines, and future studies should investigate age-related responses, timing, and long-term efficacy and safety.

## Data Availability

The original contributions presented in the study are included in the article/Supplementary material, further inquiries can be directed to the corresponding author.
