# Supplementary material for: Impact of atropine on changes in choroidal thickness in children with myopia: a meta-analysis of randomized controlled trials
Source: Front Med (Lausanne). 2025 Oct 7;12:1678698. doi: 10.3389/fmed.2025.1678698 (PMC12537749; doi:10.3389/fmed.2025.1678698)
Supplement: Supplementary file 1 [file Data_Sheet_1.PDF]

Supplementary Table 1. Characteristics of the Included Studies at Baseline in the Meta-analysis

Study	Atropine Sources and Uses	OCT inspection machine	Choroidal thickness Measurement
Yam et al 2022	Aseptic Innovative Medicine Co, LTD, Taipei, Taiwan<br>once nightly	SD-OCT (Heidelberg Engineering, Germany) with the EDI mode	MATLAB software
Kong et al 2021	Shenyang Xing qi Pharmaceutical Co, LTD, Shenyang, China<br>once nightly	SD-OCT (RS-3000, NIDEK, Co, Ltd, Japan).	built-in software
Ye et al 2020	Shenyang Xing qi Pharmaceutical Co, LTD, Shenyang, China<br>once per day	SS-OCT (Topcon Corp., Tokyo, Japan)	built-in software
Zhao et al 2021	Shenyang Xing qi Pharmaceutical Co, LTD, Shenyang, China<br>once nightly	SD-OCT (Heidelberg Engineering, Germany) with the EDI mode	Heidelberg linear measurement tool
Hao et al 2021	Shenyang Xing qi Pharmaceutical Co, LTD, Shenyang, China<br>once nightly	SD-OCT (CIRRUS HD-OCT,5000, SINGAPORE)	undescribed
Wang et al 2022	(0.05% atropine sulfate (1 ml) in poly (ethylene glycol) eye drops (4 ml))<br>once nightly	SD-OCT (Heidelberg Engineering, Germany) with the EDI mode	Heidelberg Eye Explorer software
Lee et al 2024	Undescribed<br>once nightly	SD-OCT (Heidelberg Engineering, Germany) with the EDI mode	MATLAB software

Fu et al 2024	Undescribed once nightly	SS-OCTA (VG200S; SVision Imaging, Ltd, Henan, China)	built-in software
Hansen et al 2024	Undescribed once nightly	SS-OCT	built-in software
Acquah et al 2024	Undescribed Undescribed	SS-OCT (DRI-OCT Triton Plus; Topcon Corporation, Tokyo, Japan)	MATLAB software
Zheng et al 2023	Undescribed once nightly	SD-OCT (Heidelberg Engineering, Germany)	built-in software

SD-OCT: spectral-domain optical coherence tomography; SS-OCT: swept-source optical coherence tomography

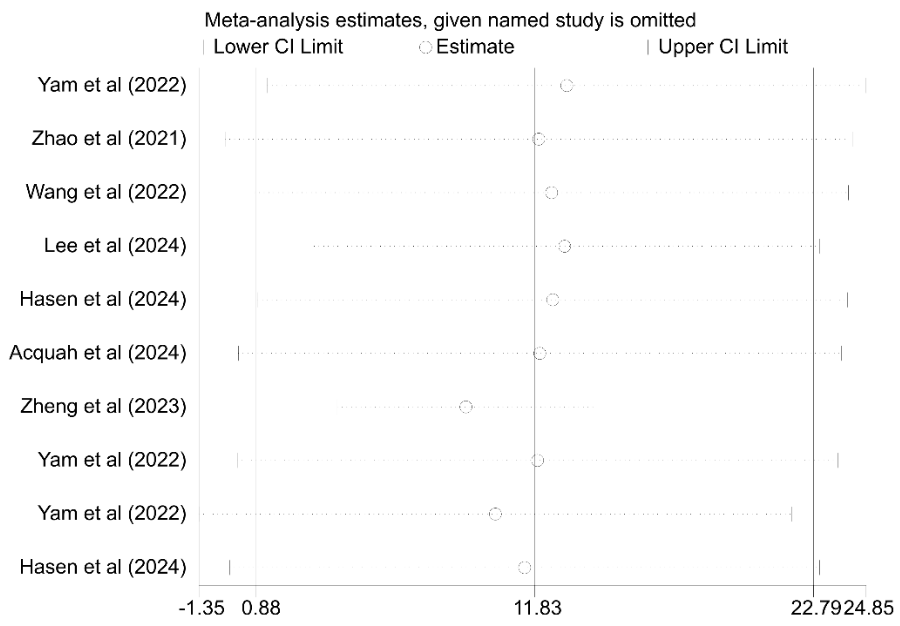

Supplementary Figure 2 sensitivity analysis of the included studies

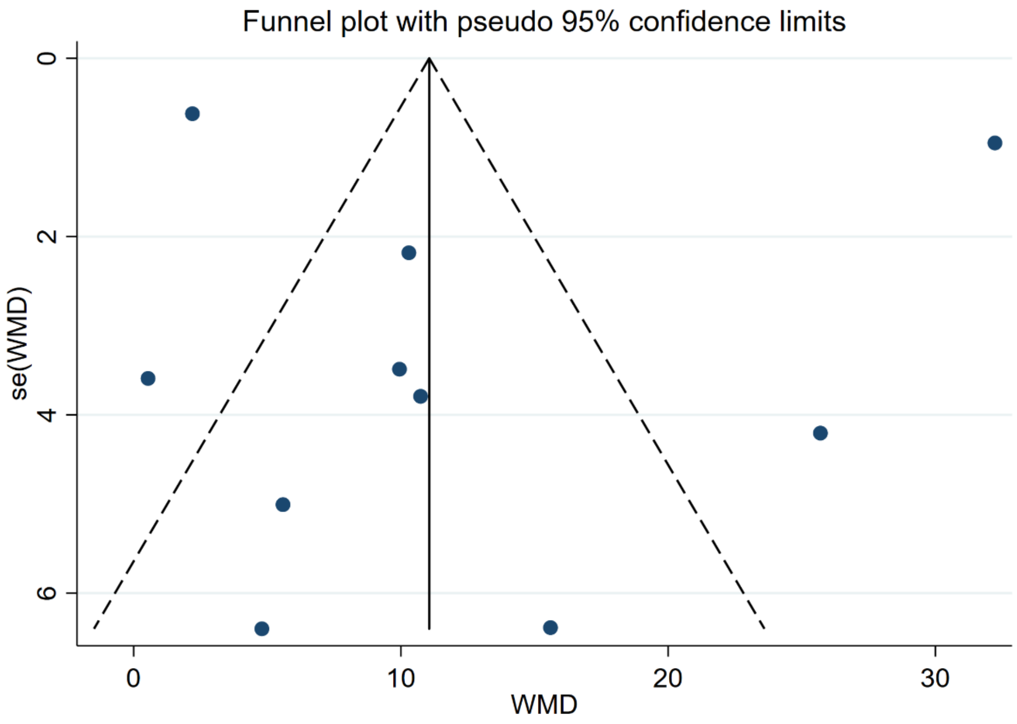

Supplementary Figure 2 funnel plot of the included studies

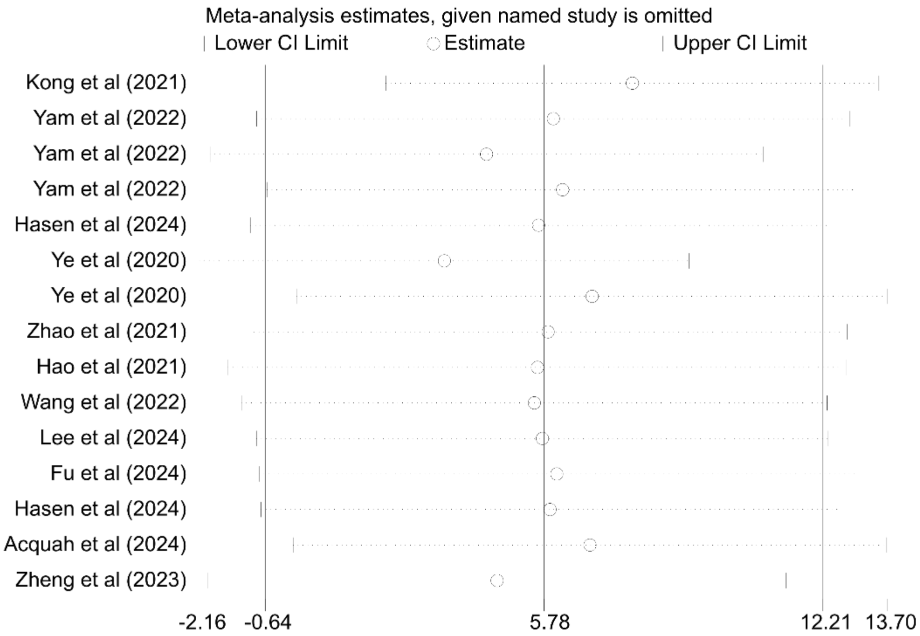

Supplementary Figure 3 sensitivity analysis of the included studies

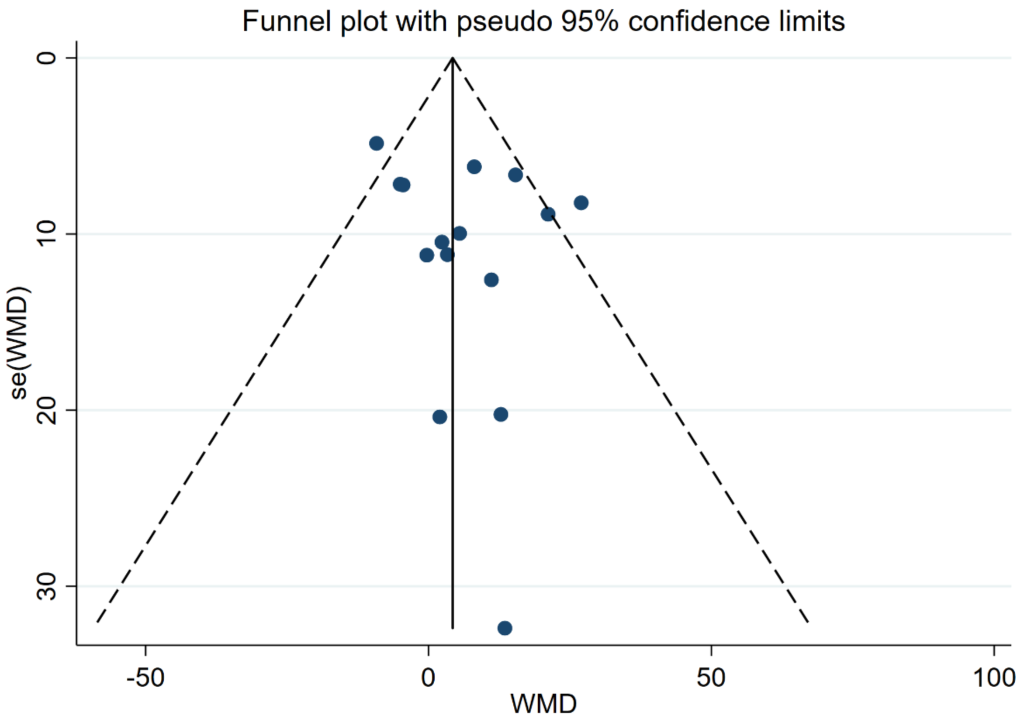

Supplementary Figure 4 funnel plot of the included studies

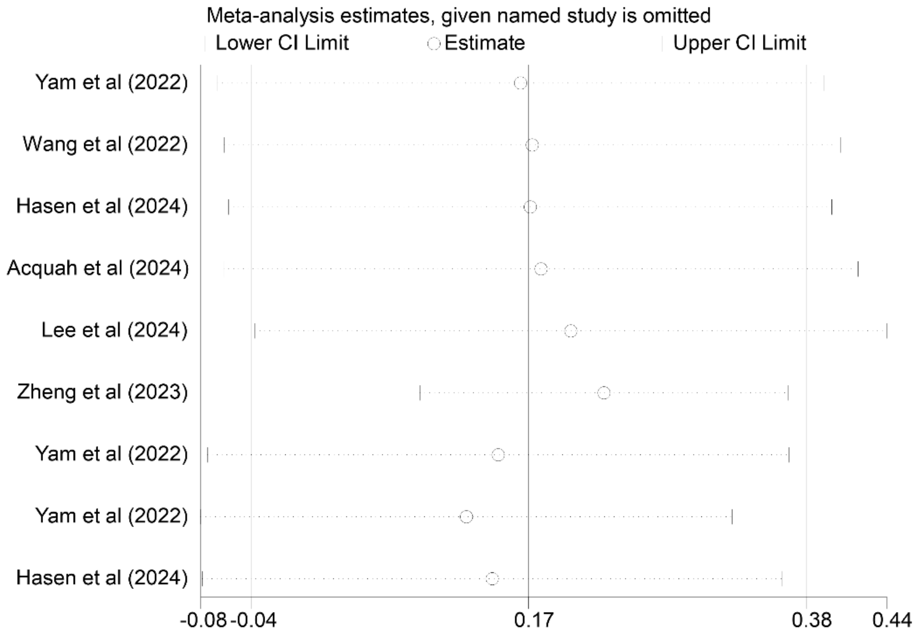

Supplementary Figure 5 sensitivity analysis of the included studies

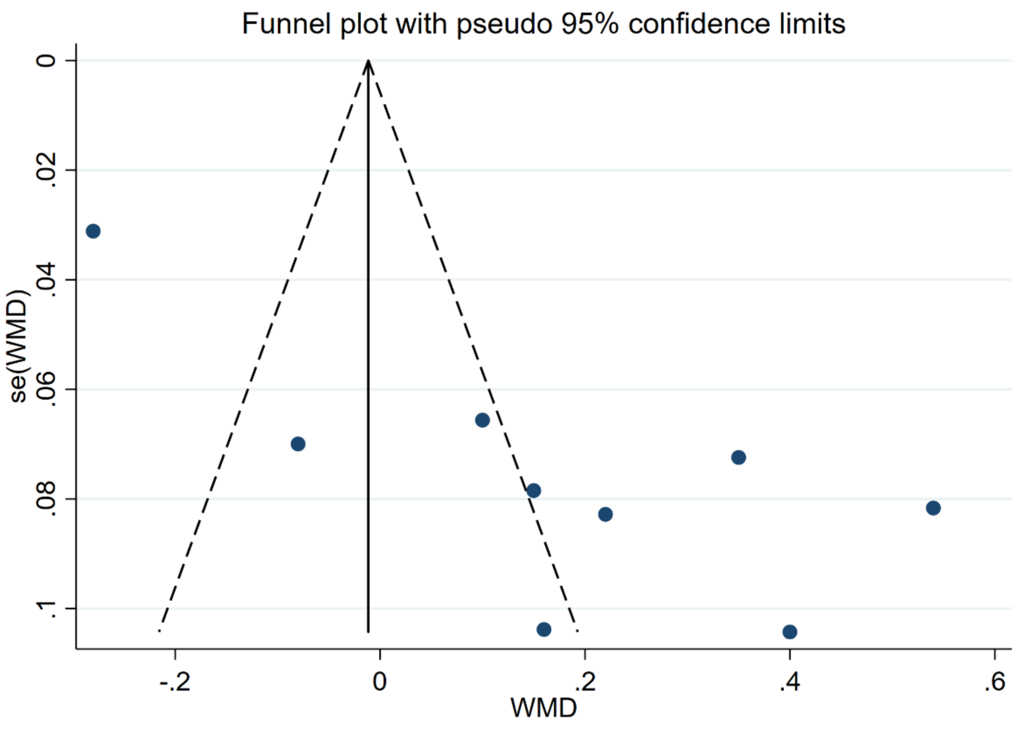

Supplementary Figure 6 funnel plot of the included studies

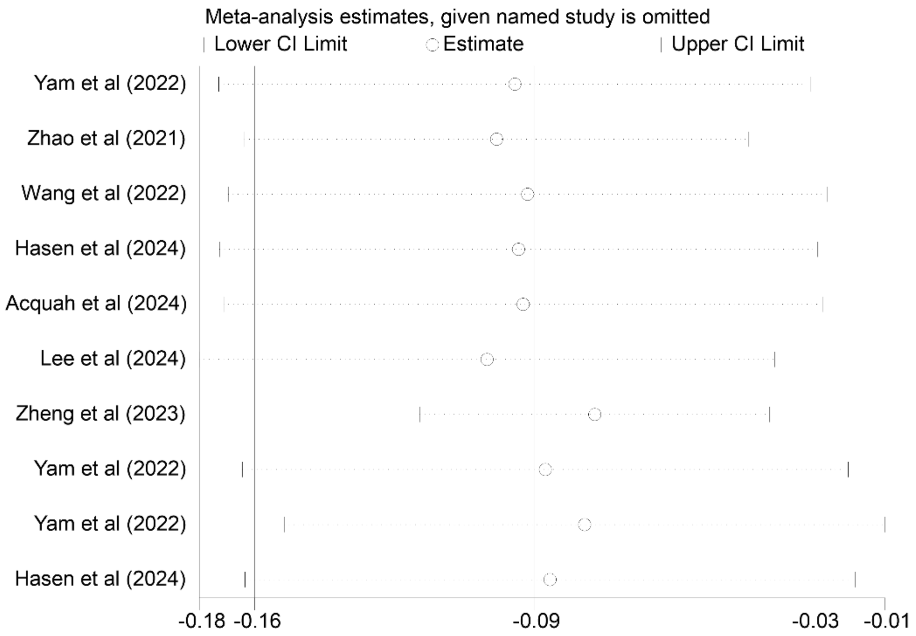

Supplementary Figure 7 **sensitivity analysis of the included studies**

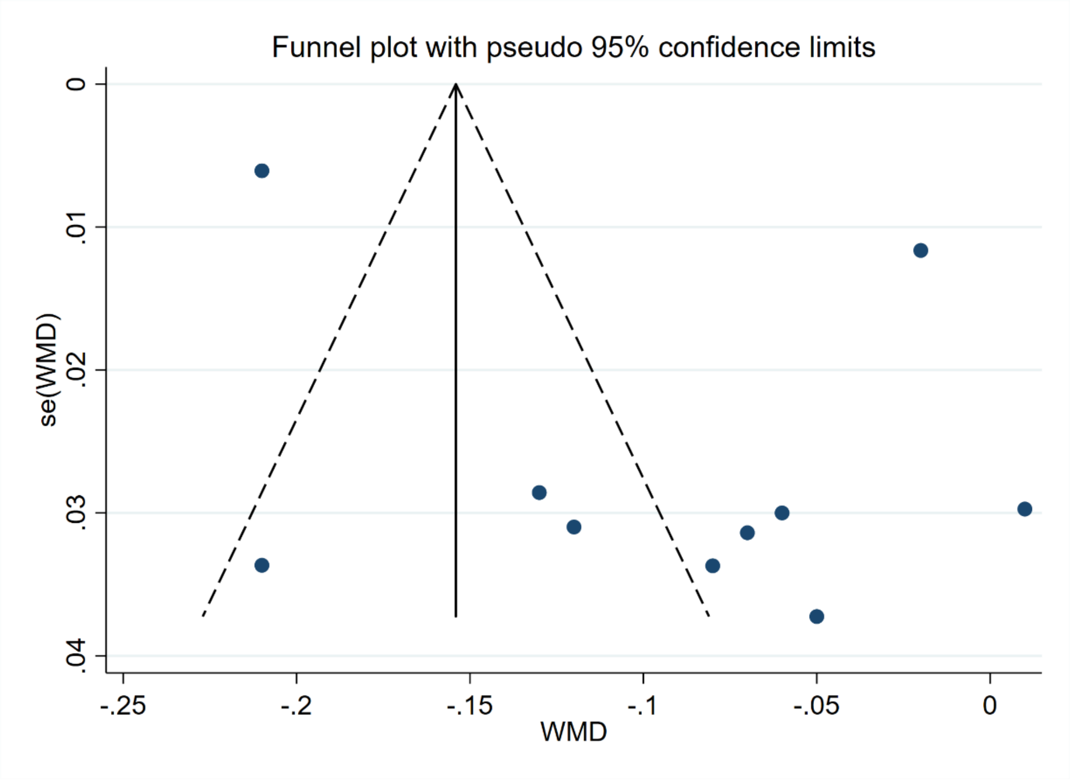

Supplementary Figure 8 **funnel plot of the included studies**
